# Bringing back the body into the mind: gestures enhance word learning in foreign language

**DOI:** 10.3389/fpsyg.2014.01467

**Published:** 2014-12-09

**Authors:** Manuela Macedonia

**Affiliations:** ^1^Information Engineering, Johannes Kepler Universität Linz, Linz, Austria; ^2^Neural Mechanisms of Human Communication, Max Planck Institute for Cognitive and Brain Sciences, Leipzig, Germany

**Keywords:** language learning, gesture, embodiment and grounded cognition, memory, education methods, brain

## Abstract

Foreign language education in the twenty-first century still teaches vocabulary mainly through reading and listening activities. This is due to the link between teaching practice and traditional philosophy of language, where language is considered to be an abstract phenomenon of the mind. However, a number of studies have shown that accompanying words or phrases of a foreign language with gestures leads to better memory results. In this paper, I review behavioral research on the positive effects of gestures on memory. Then I move to the factors that have been addressed as contributing to the effect, and I embed the reviewed evidence in the theoretical framework of embodiment. Finally, I argue that gestures accompanying foreign language vocabulary learning create embodied representations of those words. I conclude by advocating the use of gestures in future language education as a learning tool that enhances the mind.

## INTRODUCTION

In western countries, foreign language (L2) lessons employ mainly audio-visual learning (Choo et al., 2012; Graham et al., 2014). Novel vocabulary items are embedded in texts and/or missing in texts with gaps; during reading or listening, learners fill the gaps. At home, learners go through bilingual lists and read them as often as they need to in order to memorize the words (Yamamoto, 2014). As everybody has experienced, vocabulary learning is tedious and lists must be relearned regularly in order to build up the word inventory that we need for speaking.

In the 1970s Piaget noted that native language (L1) acquisition is a sensorimotor process (Piaget, 1976). Today, findings in cognitive sciences show that word learning is a process involving multisensory perception and motor acts (Tomasello, 2005; Goldin-Meadow and Alibali, 2013). In other words, word learning involves the body in cognition. Why then does language education continue to teach vocabulary mainly by hearing and reading?

The dichotomy between body and mind goes back to Descartes. In his *Discourse on the Method* (Descartes, 1637), he postulated the division between the body (with material properties and working as a machine) and what is intangible, the mind, both interacting but remaining strictly separated. In this sense, a cognitive capacity like language would be served by the body but belong to the mind. This perspective held for over two centuries. In the 1970s/1980s it was reinforced by Fodor’s influential theories (Fodor, 1976, 1987), which see language input and output as separated from the system ruling them at the base of cognitive capacity. Whereas input (hearing) and output (speaking) are sensorial, hence modal – similar to Descartes (1637), provided by the “machine” – language belongs to an abstract functional system (Pylyshyn, 1984). The rules of this system, like syntactic rules, are amodal and symbolic. Along this line of thought, semantics is also abstract: words are symbols for objects and events in the real world. Turning its back on structuralism (de Saussure, 1916), modern second language instruction found nourishing substrate in Fodor’s ideas and Chomsky’s linguistic theory (Chomsky, 1965, 1975), which again proposed language as an abstract and innate phenomenon of the mind, unrelated to the body (Ewing, 1972). With this theoretical background, second language instruction has concentrated on building up language in a similar way as it was thought that L1 acquisition occurs (Cook, 2004), i.e., by providing learners with authentic text materials (Gilmore, 2007), listening and comprehension activities (Macaro, 2006), intuitive procedures, and implicit rule acquisition (Rebuschat and Williams, 2012). Over time, it seems that memory and how to optimize vocabulary acquisition have not been considered as relevant issues in theories of language education. In spite of that, in practice, the need to acquire vocabulary has always been a core concern for teachers and learners (Coady and Huckin, 1997). This explains the large amount of vocabulary teaching materials (Davidson, 2007; Nation, 2008; Schmitt, 2008; Meara, 2009; McCarthy et al., 2010) and vocabulary games (Ghanbaran and Ketabi, 2014) published for the classroom.

## GESTURES AND MEMORY FOR WORDS AND PHRASES IN L2

The first scholar reporting about the positive effect of gestures on vocabulary retention was Radonvilliers (1768). In his book, he compared L1 with L2 learning. He noted that when explaining the word *lion* to a child in L1, an adult would show a picture or perform some illustrative gesture of the concept, whereas in L2 this does not happen. About two centuries later, Asher (1969) described the Total Physical Response approach, where learners responded to commands in L2 such as *close the door* by performing the action. Asher (1969) noticed that memory for the phrase was enhanced if learners combined action and phrase. However, Asher (1969) did not investigate his observation empirically and his work did not go beyond a theoretical position in language education. In memory research, the 1980s were a fertile decade for those research groups that tested the effect of gestures on the retention of words and phrases in the subject’s L1. The *enactment effect* (Engelkamp, 1980) or *subject performed task effect* (Cohen, 1981) was documented (Hehtrup, 1994) as robust across different populations and by different kinds of tests (see Zimmer, 2001 for a review). Note, however, that this research did not affect linguistic theory and most interestingly never reached L2 education, where, practice of vocabulary learning worked with complex elaboration of texts, flash cards and different kinds of visual learning materials in order to enhance memory for words (Clark and Paivio, 1991; Paivio, 1991). Still, the body was not taken into consideration as a learning tool.

Quinn-Allen (1995) conducted the first empirical study on the influence of gestures on memory for L2. She taught English natives short sentences in French by means of reading. For half of the sentences, subjects additionally performed cultural gestures illustrating the sentence’s semantics. Quinn-Allen (1995) found better memory results for enacted phrases in the short- and the long-term. In her doctoral dissertation, Macedonia (2003) taught German-speaking university students words of an artificial corpus audio-visually and additionally by performing a gesture. The artificial corpus was used in order to avoid associations with languages known to participants. In cued recall tests, memory performance was significantly superior for enacted items at all time points, i.e., on days 1 and 8, but also on days 15 and 73 and after 14 months. Tellier (2008) taught French pre-schoolers English words. Half of the group learned the lexical items with pictures. The other half of the group learned them by self-performing iconic gestures. Significantly better memorization was obtained through gestures. Kelly et al. (2009) worked with English natives. They learned Japanese verbs audio-visually and additionally by performing an iconic gesture. A portion of the words was accompanied by congruent gestures, the other by incongruent gestures not reflecting the word’s semantics. Congruent gestures led to better results. Macedonia et al. (2011) cued participants to accompany concrete words of an artificial corpus either with illustrative or with meaningless gestures. Memory results were significantly better for words learned with illustrative gestures in the short- and the long-term (60 days). These findings also hold for abstract words learned not isolated but embedded in sentences, as documented in a further study by Macedonia and Knösche (2011). Porter (2012) explored the effects of gestures on memory during French lessons with English children (5–7 years); two stories were told: one with pictures and one with both gestures and pictures. Again, gestures enhanced memory. Mayer et al. (2014) had participants learn novel words of an artificial corpus either by pairing them to a picture or to a gesture. Gestures could be of two kinds: iconic gestures or gestures produced by drawing the outline of the concept in the air. Compared to the baseline (reading and hearing), performing gestures was more efficient than learning with pictures. In the long-term, words learned through iconic gestures scored better than drawing their semantic shape in the air. Recently, enactment was tested with an intelligent agent as a trainer, i.e., a virtual figure with anthropomorphic appearance. The agent cued learners to perform gestures while learning words in L2 (Macedonia et al., 2014b). The first of these studies compared memory enhancement between a baseline (reading and hearing the words) and additionally performing an iconic gesture. Young adults were presented the words and the gestures either by a human or by an agent trainer. Independently of the trainer, gestures led to memory enhancement (Bergmann and Macedonia, 2013). Another study with school children enriched the audio-visual baseline by observation or observation coupled with performance of the gesture produced by a virtual agent. The results demonstrated that self-performance of the gesture is the key to enhanced learning (Macedonia et al., 2014a).

The effect of gestures on memory for words and phrases in L2 is robust and well documented. Few studies report finding no behavioral enhancement of memory (Krönke et al., 2013; Rowe et al., 2013). However, learning is a dynamic process elicited through input. The input is affected by a number of parameters that differ in most studies presented in this review. Following factors can bias results in a word-learning experiment: the phonotactic shape of the words (Baddeley et al., 1975; Gathercole and Baddeley, 1992), word familiarity (Meier et al., 2013), number of repetitions, cognitive capacities of the population (Macedonia et al., 2010), and so on. Considering the many experiments providing evidence *for* the enactment effect, the studies above might have affected these parameters in a way that enactment could not have an impact on learning performance.

## FACTORS LEADING TO MEMORY ENHANCEMENT FOR WORDS IN L2

Over the decades, the enactment effect has been explained in a number of ways. The first explanation addressed the concept of memory trace. Considering that overt performance of the gesture leads to enhancement, Engelkamp (1980) and Engelkamp and Zimmer (1985) attributed the enhancement to the creation of a motor trace. This view was confirmed many years later in neuroscientific studies documenting that audio-visual perception of words learned with gestures elicit activity in brain regions controlling motion (Masumoto et al., 2006; Macedonia et al., 2011). Considering that enactment is a multisensory process, enhancement was also attributed to the complexity of the memory trace (Tellier, 2008; Porter, 2012). This position also holds in various neuroscientific studies; for a review, see Horchak et al. (2014). Other studies explained the effect through depth of encoding (Quinn-Allen, 1995; Tellier, 2008; Kelly et al., 2009; Macedonia et al., 2011; Krönke et al., 2013; Macedonia and Klimesch, 2014), with this concept going back to Craik and Lockhart’s (1972) influential model, which suggested that information is processed at different levels, sensory information, for example hearing, being shallow and semantic processing being deep. A further factor addressed as leading to memory enhancement is mental imagery (Kelly et al., 2009; Macedonia and Knösche, 2011; Macedonia et al., 2011), where learners performing a gesture would activate an internal kinetic image of the concept/word. Support for this view comes from a review by Hostetter and Alibali (2008). There the authors propose that gestures emerge from an underlying mental image of concepts and therefore are tightly connected to them. Recently, in this line of thought, an intriguing view was presented by Straube et al. (2012), i.e., a supra-modal network in the brain serving both speech and gesture semantics.

These different approaches are not mutually exclusive. Rather, they shed light on multiple facets of enactment. On the one hand, they explain the creation of memory representation; on the other hand, they address the interconnectedness of language and gesture, hence the particular relationship between them.

## WORDS AND THE BODY

**FIGURE 1:**
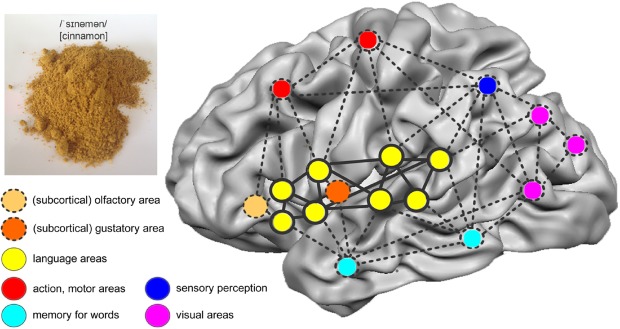
**Word network for cinnamon.** It comprises canonical language areas, areas processing and storing sensorial information experienced (visual features, odor, taste, color, texture, etc.), and motor areas involved in preparation and execution of action for manipulation and gustation of the spice.

In traditional linguistics, a word was regarded as an abstract unit of the mental lexicon (Aitchison, 1987). However, neuroscientific studies in the past decades have demonstrated that a word (in the brain’s language) is an experience-dependent functional network (Pulvermuller, 1999). This network consists of interconnected neuron assemblies in regions of the brain involved in the learning process (Kiefer and Pulvermüller, 2012; Moseley and Pulvermüller, 2014). Consider the word *cinnamon*. When a child acquires the label for the concept, i.e., the word, the child collects multisensory experience reflecting his/her interaction with the spice: olfactory and gustatory perception, consistency to the touch, visual characteristics, motor programs to interact with the spice and to articulate the word, sequence of phonemes, and so on. Through literacy, this network is enlarged by the written word (Figure 1). Neuroscientific experiments demonstrate that by activating a component of the network, other components become active and respond on stimulation. For example, mere reading of the word *cinnamon* activates the network and those brain regions processing odor and taste even if the person in the scanner cannot smell or taste cinnamon (González et al., 2006; Barros-Loscertales et al., 2012).

Similarly, verbs describing actions (such as *kick*, *lick*, and *pick*) involve representations in the brain of those body parts used to perform the action (e.g., foot, tongue, and hand; Hauk et al., 2004). Hearing the (learned) word is enough to activate brain regions that command those body parts (see Fischer and Zwaan, 2008; Watson et al., 2013, for reviews).

The principle of the network holds not only for concrete and action words, but also for abstract words. Expectedly, emotional words are strongly linked to emotional regions in the brain (Straube et al., 2011; Citron, 2012) but more interestingly to motor regions as well (Moseley et al., 2012), the latter possibly being involved during actions that accompany emotional states (Vigliocco et al., 2009). Other abstract words, depending on their emotional valence, activate an emotional network in the brain, as recently demonstrated in a functional magnetic resonance study by Vigliocco et al. (2014). Altogether, these findings provide converging evidence against symbolic theories of language. Instead, these findings show that in the brain (hence in cognition) words are represented in an experience-related way and this experience is connected to the body (Glenberg and Kaschak, 2002; Barsalou, 2008; Fischer and Zwaan, 2008).

Furthermore, being language grounded in the body and its actions, mere reading or hearing elicits simulation. Simulation, in turn, induces brain activity in those areas that are activated during physical performance.

## EMBODIMENT OF FOREIGN LANGUAGE WORDS

Understanding words in L1 reactivates experience-dependent representations in the brain (Glenberg and Gallese, 2012). Recent studies have investigated whether embodiment of language is limited to L1 or also extended to L2. Dudschig et al. (2014) hypothesized that perceiving words in L2 might also trigger motor responses similarly to L1. They presented German subjects English words such as *star* and *root* that are spatially located either above or under the subject. The subjects were asked to respond to the words with an upward or a downward arm movement. Reaction times were collected. The statistical analysis showed no differences in reaction times between native and L2. The authors take these results as evidence for the existence of sensorimotor representation for words in second language. In a reaction time experiment, Vukovic and Williams (2014) made highly bilingual Dutch subjects studying in the United Kingdom read English sentences with interlingual homophones. The task implied a distance relation. A picture followed each sentence. Subjects had to provide an answer that either matched or mismatched the distance relation. Again, the reaction time results showed that subjects, when hearing the homophones, must have simulated the word’s semantics for both L1 and L2. In a functional magnetic resonance imaging study, De Grauwe et al. (2014) made German subjects read motor verbs in Dutch, their L2. The items were subdivided in two categories: cognate (words with a similar phonetic shape and the same meaning) and non-cognate verbs. Both cognate and non-cognate verbs activated motor-related areas in the brain. These studies provide initial evidence for the existence of embodied lexical representations also in L2. The processing of such words occurs in an automatic and unconscious way, like in L1. However, *advocatus diaboli* might argue in the case of Vukovic and Williams (2014) study that results hold for highly proficient bilinguals, i.e., for learners that have collected enough sensorimotor experience for the vocabulary through full immersion. Also, cognate words might represent a special class of words with high embodiment. It is questionable whether somebody learning a new language by reading and hearing would show the same reactions when asked to perform the tasks reported in the experiment. More research is needed in order to discern how L2 words become embodied.

Here, we wonder how in L2 gestures contribute to mapping concepts into the body. In the light of the preceding sections, we presently reason that:

1. Gestures, here specifically actions, as described in Asher (1969), performed during novel L2-word learning (e.g., German *gehen*, English *to go*, by English learners) connect to a pre-existing embodied representation(s) in the learners’ L1. Also, it is possible that *gehen* creates its own sensorimotor representation in a similar way as in L1;
2. In the case of a word that cannot be represented by an action (e.g., English *bridge* or *thought)*, an illustrative (iconic) gesture might match an internal kinetic image of the word previously created in L1, therefore connect the L2 word and the embodied representation on a more abstract level. The existence of an internal image applying also for L2 has been demonstrated in a brain imaging study. In this study, upon word recognition, incongruent gestures performed to L2 words elicited a Stroop-task-like network in the brain denoting disturbance (Macedonia et al., 2011);
3. For function words (e.g., *already* or *although*), gestures can only be symbolic and arbitrary, as proposed by Macedonia and Knösche (2011). In that study, gestures enhanced learning. However, it still has to be demonstrated whether these gestures do create a novel embodied representation in L1, as those words are highly abstract.

More empirical research is needed in order to turn speculations into knowledge. However, the foundation of a new vision of language instruction grounded in the learner’s body has been laid.

## CONCLUSION

In the past two decades, amodal theories of language have been massively challenged through progress in neuroscience. Empirical evidence has shown that language learning and representation are intrinsically connected to the body. This evidence has given birth to various theories of embodiment that are still being discussed in the light of empirical findings (see Meteyard et al., 2012; Horchak et al., 2014, for reviews). Independently of this, embodiment is giving language education a cutting edge by authorizing it to consider the body as a learning tool. In the future this will hopefully enable learners to exploit natural and L1-like strategies and to improve L2 word acquisition (Macedonia, 2013). In a few years we will have a more comprehensive picture of language as an embodied cognitive capacity. Recent research also considers that language needs both the body but also abstract symbols (Arbib et al., 2014). However, after decades of symbolism, it is the body’s turn in L2 vocabulary learning!

### Conflict of Interest Statement

The author declares that the research was conducted in the absence of any commercial or financial relationships that could be construed as a potential conflict of interest.
